# Most elite athletes return to preinjury competitive activity after surgical treatment for medial malleolus stress fractures

**DOI:** 10.1002/ksa.12284

**Published:** 2024-06-03

**Authors:** Kishan R. Ramsodit, Ruben Zwiers, Miki Dalmau‐Pastor, Vincent Gouttebarge, Gino M. M. J. Kerkhoffs

**Affiliations:** ^1^ Amsterdam UMC location University of Amsterdam Department of Orthopedic Surgery and Sports Medicine, Meibergdreef 9 Amsterdam The Netherlands; ^2^ Academic Center for Evidence Based Sports Medicine (ACES) Amsterdam The Netherlands; ^3^ Amsterdam Collaboration on Health and Safety in Sports (ACHSS), IOC Research Center of Excellence Amsterdam The Netherlands; ^4^ Amsterdam Movement Sciences (AMS), Aging & Vitality, Musculoskeletal Health, Sports Amsterdam The Netherlands; ^5^ Department of Orthopedic Surgery Flevoziekenhuis Almere The Netherlands; ^6^ Human Anatomy and Embryology Unit, Department of Pathology and Experimental Therapeutics, School of Medicine and Health Sciences The University of Barcelona Barcelona Spain; ^7^ MIFAS by GRECMIP (Minimally Invasive Foot and Ankle Society) Merignac France; ^8^ Section Sports Medicine, Faculty of Health Sciences University of Pretoria Pretoria South Africa

**Keywords:** elite athlete, medial malleolus stress fracture, return to performance, stress fracture

## Abstract

**Purpose:**

To provide return‐to‐performance outcomes after surgical treatment for medial malleolus stress fractures in the elite athlete. Additionally, to describe an individualised surgical approach in the management of medial malleolus stress fractures.

**Methods:**

Five athletes (six ankles) underwent surgical treatment for a medial malleolus stress fracture. The surgical technique was based on the extent of the fracture line in steps with first arthroscopic debridement of bony spurs, microfracturing of the fracture line and screw fixation. Return‐to‐performance data included time to return to sport‐specific training, normal training, first competitive activity, performance and the return‐to‐performance rate.

**Results:**

Patients returned to sport‐specific training at a median of 10 weeks. They started normal training at 16 weeks postoperatively and returned to their first competitive activity after 19 weeks. All patients had bony spurs on the distal tibia which were arthroscopically debrided. One patient received arthroscopic debridement of bony spurs alone. Four patients received additional microfracturing of the fracture line and three patients received screw fixation. All patients achieved clinical and radiographic union on follow‐up computed tomography scan at 3 months postsurgery. At latest follow‐up, no refractures nor hardware complications, nor any other complications were observed.

**Conclusion:**

Arthroscopic debridement of bony spurs, debridement and microfracturing of the fracture line and screw fixation are all viable surgical tools in the management of medial malleolus stress fractures in elite athletes. The surgical approach containing these options should be tailored to the individual athlete based on the fracture line in the sagittal plane. While most athletes return to full competitive activity in 3–4 months, time to self‐reported return to full performance is often much longer.

**Level of Evidence:**

Level IV.

**Abbreviations**
AiTFL: anterior inferior tibiofibular ligament
ATFL: anterior talofibular ligament
CLAI: chronic lateral ankle instability
CT: computed tomography
IQR: interquartile range
MRI: magnetic resonance imaging
NWB: nonweight‐bearing
ORIF: open reduction internal fixation
PWB: partial weight‐bearing
ROM: range of motion
WB: weight bearing

## INTRODUCTION

Stress fractures, while uncommon in the general population, are frequently seen in the athletic population [3, 4]. Medial malleolus stress fractures are devastating injuries to athletes and classified as high‐risk stress fractures due to a high risk of delayed union, nonunion and progression to a complete fracture [5, 16]. While their aetiology is thought to be comparable to other stress fractures, it is theorised that anterior bony spurs of the distal tibia and/or talus and varus alignment are risk factors for the development of medial malleolus stress fractures [9, 12]. Medial malleolar stress fractures are a rare cause of medial ankle pain, occurring almost exclusively in elite athletes engaged in repeated running and jumping sporting activities [14, 17]. The patient typically presents with a gradual onset of medial ankle pain along with tenderness over the medial malleolus [15]. However, a high index suspicion of this injury must be present for the diagnosis due to its rare nature and therefore this injury can easily be missed or misdiagnosed. This results in the delay of adequate treatment and can impede the return to performance of an athlete. Conservative treatment has shown to be a successful treatment option; however, for the elite athlete, surgical intervention is often preferred due to a higher union rate and quicker return to previous sports activity [11, 12, 14]. Even though this injury is most often seen in athletes, time to return to performance is uncertain as no strict definitions and criteria are used to assess return‐to‐performance outcomes. This emphasises the need for more detailed data on return to performance outcomes after surgical treatment for such a rare injury.

Hence, this study presents a series of five athletes with the aim of providing an overview of the clinical course and return‐to‐sport process after surgical treatment for a medial malleolus stress fracture in elite athlete. Additionally, this study aims to provide a description of an individualised approach for the surgical treatment of medial malleolus stress fractures.

## MATERIALS AND METHODS

### Patient selection

Elite athletes, defined as competing professionally at a national or international level of competition at the time of injury, were identified through electronic patient records. Elite athletes with a surgically treated medial malleolus stress fracture were included in this study. Athletes who suffered an injury unrelated to the surgery during their rehabilitation were excluded.

### Anatomy and radiological imaging

**Figure 1:**
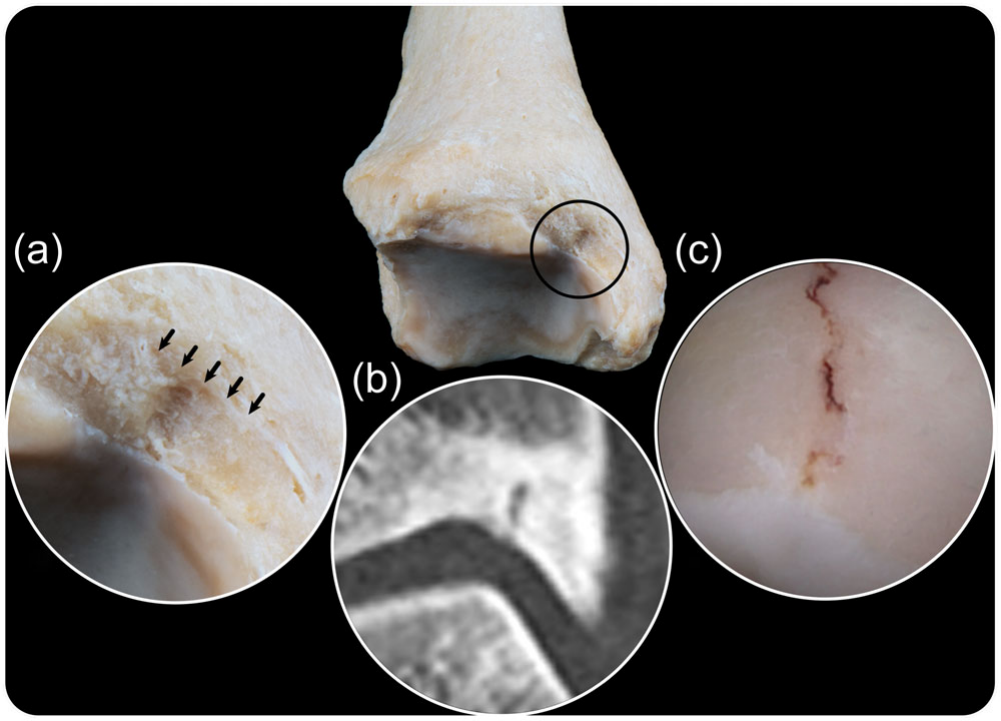
Image showing the anatomy of the distal tibia, including the medial malleolus and Harty's notch. (a) Magnification of the bony anatomy with Harty's notch highlighted (black arrows). (b) Computed tomography scan of a patient with a medial malleolus stress fracture. (c) Arthroscopic image of a medial malleolus stress fracture.

All patients received a computed tomography (CT) and magnetic resonance imaging (MRI) scan prior to surgery. A CT scan was the preferred imaging modality in case of suspicion of a medial malleolus stress fracture. It shows the characteristic osteophytes and bony spurs alongside sclerosis of the anteromedial distal tibia. Additionally, the representative vertical or oblique fracture line, either complete or incomplete, arising from the junction of the medial malleolus and tibial plafond can be observed (Figure 1b,c). Incomplete fractures were defined as a singular breach in the cortex of the anteromedial tibial plafond. Figure 1a also illustrates Harty's notch, an anatomic variant and pseudodefect of the tibial plafond, which has the same location as the medial malleolus stress fractures. An MRI scan has the advantage of visualising the bone marrow activity in case of stress reactions, while ruling out other and concomitant pathology.

### Surgical technique

**Figure 2:**
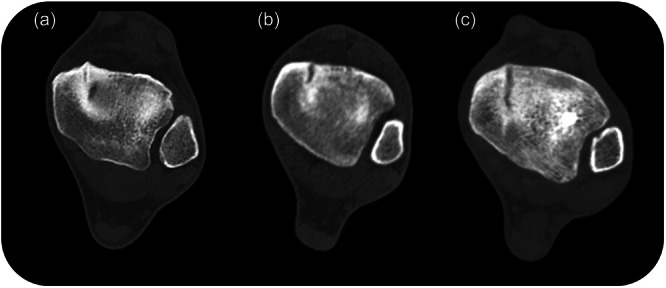
Axial view of computed tomography scans of the ankle with a medial malleolus stress fracture in various stages. (a) Stress fracture confined to the anteromedial osteophyte; (b) stress fracture progressing beyond the anteromedial osteophyte; (c) stress fracture encompassing 50% of the medial malleolus in the sagittal plane.

**Figure 3:**
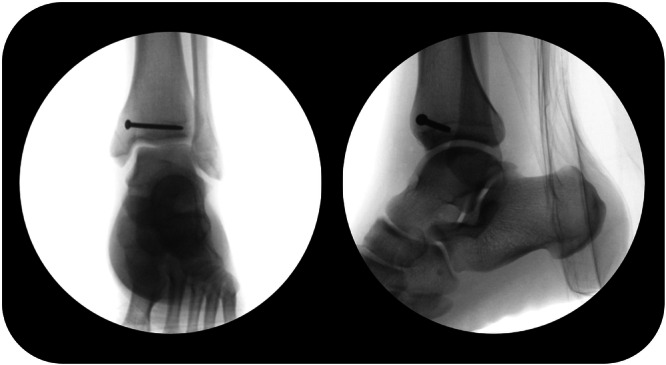
Anterior–posterior and lateral radiograph of the ankle showing screw fixation of the fracture.

All surgeries were performed by an experienced orthopaedic surgeon, specialised in foot and ankle sports injuries (G. M. M. J. K). The surgical technique consisted of arthroscopic spur resection and debridement with or without bone marrow stimulation and screw fixation. Ankle arthroscopy was performed to visually assess the ankle joint for other pathology and identify the fracture line or bony spurs. Bony spurs, sclerotic bone and the fissure were debrided until the visible cancellous bone was observed. If the fracture line was visible after arthroscopic spur resection and fracture line debridement, bone marrow stimulation was performed. Using an arthroscopic microfracture pick, small holes were made in the bone surrounding the fissure until subchondral bleeding was visualised. If the fracture line progressed further than 50% of the medial malleolus in the sagittal plane, percutaneous screw fixation was performed (Figure 2). Using fluoroscopy, the entry point for the screw was localised, just above the ankle joint. Subsequently, a stab incision was made over the medial malleolus and a 3.5 mm cortical screw was placed perpendicularly to the fracture line under fluoroscopy guidance (Figure 3). If necessary, a second screw was placed to achieve sufficient compression over the fracture. Finally, the ankle arthroscopy was used to assess if fissure compression was achieved.

### Postoperative rehabilitation protocol

Two different postoperative rehabilitation protocols were used, depending on whether screw fixation was performed. If no screw fixation was performed, a nonimmobilisation protocol was followed in which patients received a pressure bandage for 3 days and used elbow crutches for 2 weeks. Immediately postsurgery, they were encouraged to start range of motion (ROM) exercises of the ankle. In case of screw fixation, an immobilisation protocol was used during which the ankle was immobilised for 2 weeks in a nonweight‐bearing (NWB) cast, 2 weeks in a partial weight‐bearing cast and 2 weeks weight‐bearing in a walking boot. During the NWB cast, immobilisation patients received thromboprophylaxis with rivaroxaban 10 mg once a day. Once patients received the walking boot, they were encouraged to start training the ROM of the ankle and regain ankle strength and stability. When full ROM and sufficient strength and stability were achieved, the focus was switched to regaining the normal gait. At the time of clinical union, defined as no palpable tenderness over the medial malleolus and anteromedial corner of the distal tibia, patients were allowed to increase their training volume.

### Outcome measures

The primary outcome measure in this study was the time to return to performance. Performance was defined as self‐reported preinjury level of sport. The return‐to‐sport outcomes were defined according to definitions reported in a 2016 consensus statement [1]. Outcomes included return to training, return to sport and return to performance. These three milestones were further divided into subcategories to provide a more nuanced and complete overview of the return‐to‐sport process. Return to training included time to return to sport‐specific training, time to return to normal training and time to return to unrestricted training. Return to sport included time to return to first competitive activity, time to first full competitive activity. All time to event outcome measures were reported in weeks. Additionally, union rate, refracture rate and postoperative complications were included as clinical outcomes. In case microfracturing or screw fixation was performed, patients received a CT scan at 3 months postoperatively to assess radiographic union while clinical union was assessed during physical examination.

### Data collection

Demographic data, injury characteristics, intraoperative findings, clinical outcomes and return‐to‐performance data were extracted from patient records and surgical reports. Demographic information consisted of age, gender, sport, level of participation and player position of athlete characteristics (if applicable). Injury characteristics included injury date, time from onset of symptoms to presentation and laterality. To retrieve return‐to‐performance data, the patients were sent a questionnaire. To maximise response rate, a reminder was sent after 1 and 2 weeks.

### Statistical analysis

Descriptive statistics were used to report population characteristics. Due to the small sample size, continuous variables were reported as median with the corresponding interquartile ranges (IQRs). Categorical variables were reported as frequencies with accompanying percentages. All statistical analysis in this study was conducted using SPSS 27.0 (IBM)

## RESULTS

The present study included five athletes with a median age of 20 (IQR: 17–25) years (Table 1). The median follow‐up was 4 (IQR: 3–6) years. At the latest follow‐up, no refractures nor complications were observed. Median duration of symptoms until the presentation was 30 days (IQR: 14–70). One athlete had a bilateral medial malleolus stress fracture. All athletes had bony spurs on the distal tibia which were arthroscopically resected and debrided. One of the athletes (case 5) received arthroscopic resection and debridement of bony spurs alone as the stress fracture did not extend further than the osteophyte. Four athletes underwent microfracturing and in three athletes additional screw fixation of the fracture was performed. Four out of the five athletes returned to performance (Table 1). Athletes returned to sport‐specific training at a median of 10 (IQR: 10–13) weeks. They started normal training at 16 (IQR: 13–23) weeks postoperatively and returned to their first competitive activity after 19 (IQR: 15–33) weeks. They returned to full competitive activity at a median of 22 (IQR: 18–49) weeks.

**Table 1:** Overview of patient, injury, treatment and return‐to‐performance characteristics.

Case	Sex	Age at surgery, years	Preinjury sports	Duration of symptoms, *n* days	Surgical treatment	Rehabilitation protocol	Return to sport‐specific training, *n* weeks	Return to normal training, *n* weeks	Return to unrestricted training, *n* weeks	Return to first competitive activity, *n* weeks	Return to full competitive activity, *n* weeks	Return to performance, *n* weeks
1	M	20	Professional football player	14	Arthroscopic debridement, microfracturing and screw fixation	Immobilisation	10	18	18	19	25	25
2^a^	M	20	Professional football player	30	Arthroscopic debridement, microfracturing and screw fixation	Immobilisation	14	29	43	47	73	N/A
3	M	26	Professional football player	10	Arthroscopic debridement, microfracturing and screw fixation	Immobilisation	10	14	N/A	18	20	50
4	M	23	Professional basketball player	30	Arthroscopic debridement and microfracturing	Nonimmobilisation	10	11	13	12	15	15
5	M	30	International rugby player	50	Arthroscopic debridement of osteophytes and stress fracture	Nonimmobilisation	12	16	20	18	22	45

Abbreviation: FU, follow‐up.

^a^ Bilateral medial malleolus stress fracture.

## DISCUSSION

The most important finding of this study was that an individualised surgical approach containing arthroscopic spur debridement with optional microfracturing of the fracture line and screw fixation showed good return‐to‐sport outcomes in elite athletes. All patients returned to their respective sports and are still competing competitively to this date. Additionally, at a median follow‐up of 4 years, no refractures or hardware complications had occurred.

### Return to sport

To our knowledge, this is the first study reporting a comprehensive return‐to‐performance timeframe with defined outcomes after surgical treatment for medial malleolus stress fractures in elite athletes. Return‐to‐sport outcomes have been reported, however, the definitions of these outcomes varied. This study shows that being able to play full matches is not similar to self‐reported full performance. In a previous study, professional football players returned to full activities after 3.7 months and returned to professional football 4 months after surgery [12]. However, no clear definitions for these outcomes were provided. The two professional football players with a unilateral medial malleolus stress fracture in our study returned to normal training within 14 and 18 weeks, respectively. They played their first match after 18 and 19 weeks, respectively. The figure skater in the present study did not return to performance as she suffered an unrelated ankle injury during rehabilitation which required surgical treatment and chose to discontinue her professional career. The professional football player with a bilateral medial malleolus stress fracture returned to sport‐specific training after 14 weeks, however, his return to full competitive activity, occurring at 47 weeks postsurgery, was longer compared to the unilateral cases.

### Surgical technique

Open reduction internal fixation (ORIF) is the most common approach for the surgical treatment of medial malleolus stress fractures with some variations regarding the number of screws used [8]. An ORIF with three screws and the addition of concentrated bone marrow aspirate provided excellent results in a population of professional soccer players [12]. The present study used a percutaneous screw fixation technique with either one or two screws and achieved union in all cases with no observed refractures. Drilling of the fracture line has been previously reported to be successful as to stimulate bone healing to achieve union in patients with delayed healing of medial malleolus stress fractures [13]. In our study, microfracturing was performed in all cases with a visible fracture following arthroscopic spur debridement to stimulate bone healing. One patient received arthroscopic spur debridement and microfracturing without screw fixation.

### Contributing factors

Various potential factors contributing to the development of medial malleolus stress fractures have been discussed in the literature. These included chronic anteromedial ankle impingement, lower limb varus alignment and chronic lateral ankle instability (CLAI) [9, 10, 12]. Chronic ankle impingement was observed in all athletes in the current study, while three athletes had a history of recurrent ankle sprains.

CLAI, chronic anteromedial ankle impingement and lower limb varus malalignment complement the injury development as the anteromedial talar edge can repeatedly collide with the anteromedial bony spur of the distal tibia during high‐load supination and/or dorsiflexion of the ankle causing sclerotic bone formation as a defence mechanism of the bone. If a certain threshold is passed, a stress reaction occurs starting in the bony spur. Submaximal loading of the bone over time leads to microfractures which cannot heal due to imbalances in bone resorption and formation with further unrestricted activity [19]. This stress reaction in the sclerotic bone can then potentially be overloaded and progress further into the healthy bone of the medial malleolus [5].

Interestingly, all the football players in this study trained and played matches on artificial turf which is thought to have a higher peak torque and rotational stiffness compared to natural grass [18]. Friction forces between the turf and foot are then potentially increased, which increases the chance of injuries [7]. The ankle ROM, particularly dorsiflexion, is another potential contributing factor of this stress fracture as the ankle joint endures more of the load and reaction forces from the ground with limited dorsiflexion. The second case of this study is an example of various contributing factors coming together. The athlete had chronic anteromedial ankle impingement, varus malalignment, a limited ankle dorsiflexion and he suffered the injury during his first season on artificial turf.

Medial malleolus stress fractures are typically located at the junction of the medial malleolus and the distal tibia. While radiological imaging is advised to confirm diagnosis, the literature is not consistent in the imaging modalities used. Use of CT and MRI scans have both been reported and in the current study, patients routinely received both a CT and MRI scan. When evaluating a medial malleolus stress fracture on CT or MRI, it is important to differentiate a stress reaction or beginning stress fracture with the normal image of the Harty's notch. The Harty's notch is an anatomic variant present in up to 45% of cases, also known as a pseudodefect of the tibial plafond [2, 6]. Remarkably, the Harty's notch and the stress fractures found in this study lie in the same location (Figure 1). However, no previous studies have reported a correlation between Harty's notch and a medial malleolus stress fracture, and none of the patients in the current study had a Harty's notch.

The main limitation of the present study was the small sample size and the retrospective design which is, therefore, subject to the known limitations. Due to the small sample size, it was not possible to compare the results between the different surgical approaches. Additionally, measuring return to performance as a self‐reported outcome measure is limited by its subjective nature as this outcome can be influenced by other factors and cannot be objectively compared to other patients. The present study finds its strength in the method of return to sport data collection and the strict definition used regarding the return to performance outcome terms. This is the first study to describe the return‐to‐performance process in detail after surgical treatment for medial malleolus stress fractures in elite athletes.

## CONCLUSION

Arthroscopic debridement of bony spurs, debridement and microfracturing of the fracture line and screw fixation are all viable surgical tools in the management of medial malleolus stress fractures in elite athletes. The surgical approach containing these options should be tailored to the individual athlete based on the fracture line in the sagittal plane. While most athletes return to full competitive activity in 3–4 months, time to self‐reported return to full performance is often much longer.

## AUTHOR CONTRIBUTIONS

Kishan R. Ramsodit and Ruben Zwiers have made substantial contributions to conception and design, acquisition of data, analysis and interpretation of data and have been involved in drafting the manuscripts. Miki Dalmau‐Pastor, Vincent Gouttebarge and Gino M. M. J. Kerkhoffs have been involved in revising the manuscript, critically for important intellectual content and have given final approval of the version to be published.

## CONFLICT OF INTEREST STATEMENT

The authors declare no conflict of interest.

## ETHICS STATEMENT

The present study is a retrospective case series of consecutive elite athletes who underwent surgical treatment for medial malleolus stress fractures. Ethics approval for this study was provided by the Medical Ethical Committee of Amsterdam UMC (W22_397). Informed consent was obtained from each patient prior to participation in this study.

## References

[1] Ardern, C.L. , Glasgow, P. , Schneiders, A. , Witvrouw, E. , Clarsen, B. , Cools, A. et al. (2016) 2016 Consensus statement on return to sport from the First World Congress in Sports Physical Therapy, Bern. British Journal of Sports Medicine, 50, 853–864. Available from: 10.1136/bjsports-2016-096278 27226389

[2] Arndt, H. , German, T. , Fischer, W. , Rehnitz, C. , Antón Jiménez, A. & Weber, M.A. (2024) MRI identification of pseudolesions in the distal tibia articular surface: frequency and diagnostic criteria. European Journal of Radiology, 170, 111234. Available from: 10.1016/j.ejrad.2023.111234 38042021

[3] Bennell, K.L. , Malcolm, S.A. , Thomas, S.A. , Reid, S.J. , Brukner, P.D. , Ebeling, P.R. et al. (1996) Risk factors for stress fractures in track and field athletes: a twelve‐month prospective study. The American Journal of Sports Medicine, 24, 810–818. Available from: 10.1177/036354659602400617 8947404

[4] Bennell, K. , Matheson, G. , Meeuwisse, W. & Brukner, P. (1999) Risk factors for stress fractures. Sports Medicine, 28, 91–122. Available from: 10.2165/00007256-199928020-00004 10492029

[5] Boden, B.P. & Osbahr, D.C. (2000) High‐risk stress fractures: evaluation and treatment. Journal of the American Academy of Orthopaedic Surgeons, 8, 344–353. Available from: 10.5435/00124635-200011000-00002 11104398

[6] Boutin, R.D. , Chang, J. , Bateni, C. , Giza, E. , Wisner, E.R. & Yao, L. (2015) The Notch of harty (pseudodefect of the tibial plafond): frequency and characteristic findings at MRI of the ankle. American Journal of Roentgenology, 205, 358–363. Available from: 10.2214/AJR.14.14012 26204288

[7] Gould, H.P. , Lostetter, S.J. , Samuelson, E.R. & Guyton, G.P. (2023) Lower extremity injury rates on artificial turf versus natural grass playing surfaces: a systematic review. The American Journal of Sports Medicine, 51, 1615–1621. Available from: 10.1177/03635465211069562 35593739

[8] Irion, V. , Miller, T.L. & Kaeding, C.C. (2014) The treatment and outcomes of medial malleolar stress fractures: a systematic review of the literature. Sports Health: A Multidisciplinary Approach, 6, 527–530. Available from: 10.1177/1941738114546089 PMC4212354 25364485

[9] Jowett, A.J.L. , Birks, C.L. & Blackney, M.C. (2008) Medial malleolar stress fracture secondary to chronic ankle impingement. Foot & Ankle International, 29, 716–721. Available from: 10.3113/FAI.2008.0716 18785422

[10] Lee, H.S. , Lee, Y.K. , Kim, H.S. , Lee, D.W. , Won, S.H. , Jung, K.J. et al. (2019) Medial malleolar stress fracture resulting from repetitive stress caused by lateral ankle instability: a case report. Medicine, 98, e14311. Available from: 10.1097/MD.0000000000014311 30702607 PMC6380793

[11] Lempainen, L. , Liimatainen, E. , Heikkilä, J. , Alonso, J. , Sarimo, J. , Mattila, K. et al. (2012) Medial malleolar stress fracture in athletes: diagnosis and operative treatment. Scandinavian Journal of Surgery, 101, 261–264. Available from: 10.1177/145749691210100407 23238501

[12] Nguyen, A. , Beasley, I. & Calder, J. (2019) Stress fractures of the medial malleolus in the professional soccer player demonstrate excellent outcomes when treated with open reduction internal fixation and arthroscopic spur debridement. Knee Surgery, Sports Traumatology, Arthroscopy, 27, 2884–2889. Available from: 10.1007/s00167-019-05483-6 30915513

[13] Orava, S. , Karpakka, J. , Taimela, S. , Hulkko, A. , Permi, J. & Kujala, U. (1995) Stress fracture of the medial malleolus. The Journal of Bone & Joint Surgery, 77, 362–365. Available from: 10.2106/00004623-199503000-00005 7890784

[14] Shabat, S. , Sampson, K.B. , Mann, G. , Gepstein, R. , Eliakim, A. , Shenkman, Z. et al. (2002) Stress fractures of the medial malleolus—review of the literature and report of a 15‐year‐old elite gymnast. Foot & Ankle International, 23, 647–650. Available from: 10.1177/107110070202300711 12146777

[15] Shelbourne, K.D. , Fisher, D.A. , Rettig, A.C. & McCarroll, J.R. (1988) Stress fractures of the medial malleolus. The American Journal of Sports Medicine, 16, 60–63. Available from: 10.1177/036354658801600111 3344882

[16] Sherbondy, P.S. & Sebastianelli, W.J. (2006) Stress fractures of the medial malleolus and distal fibula. Clinics in Sports Medicine, 25, 129–137. Available from: 10.1016/j.csm.2005.08.006 16324979

[17] van den Bekerom, M.P.J. , Kerkhoffs, G.M.M.J. & van Dijk, C.N. (2009) Treatment of medial malleolar stress fractures. Operative Techniques in Sports Medicine, 17, 106–111. Available from: 10.1053/j.otsm.2009.05.006

[18] Villwock, M.R. , Meyer, E.G. , Powell, J.W. , Fouty, A.J. & Haut, R.C. (2009) Football playing surface and shoe design affect rotational traction. The American Journal of Sports Medicine, 37, 518–525. Available from: 10.1177/0363546508328108 19168808

[19] Welck, M.J. , Hayes, T. , Pastides, P. , Khan, W. & Rudge, B. (2017) Stress fractures of the foot and ankle. Injury, 48, 1722–1726. Available from: 10.1016/j.injury.2015.06.015 26412591

